# Metabotropic glutamate receptor 5 tracer [^18^F]-FPEB displays increased binding potential in postcentral gyrus and cerebellum of male individuals with autism: a pilot PET study

**DOI:** 10.1186/s40673-018-0082-1

**Published:** 2018-02-12

**Authors:** S. Hossein Fatemi, Dean F. Wong, James R. Brašić, Hiroto Kuwabara, Anil Mathur, Timothy D. Folsom, Suma Jacob, George M. Realmuto, José V. Pardo, Susanne Lee

**Affiliations:** 1 Department of Psychiatry, Division of Neuroscience Research, University of Minnesota Medical School, 420 Delaware St SE, MMC 392, Minneapolis, MN 55455 USA; 2 Department of Neuroscience, University of Minnesota Medical School, 321 Church St. SE, Minneapolis, MN 55455 USA; 3 The Russell H. Morgan Department of Radiology and Radiological Science, Section of High Resolution Brain PET Imaging, Division of Nuclear Medicine, and Molecular Imaging, The Johns Hopkins School of Medicine, Johns Hopkins Medical Institutions, Baltimore, MD USA; 4 Department of Neurology, Johns Hopkins University School of Medicine, Johns Hopkins University, Baltimore, MD USA; 5 Department of Psychiatry and Behavioral Sciences, Johns Hopkins University School of Medicine, Baltimore, MD USA; 6 Department of Neuroscience, Johns Hopkins University, Baltimore, MD USA; 7 Department of Psychiatry, Veterans Affairs Medical Center, 1 Veterans Drive, Minneapolis, MN 55417-2399 USA

**Keywords:** PET, Autism, mGluR5, Cerebellum, Postcentral gyrus, Precuneus, [^18^F]-3-fluoro-5-[(pyridin-3-yl)ethynyl]benzonitrile

## Abstract

**Background:**

Autism is a neurodevelopmental disorder that is first manifested during early childhood. Postmortem experiments have identified significantly elevated expression of metabotropic glutamate receptor 5 (mGluR5) in cerebellar vermis and prefrontal cortex of individuals with autism.

**Methods:**

In the current study we employed the mGluR5 tracer [^18^F]-3-fluoro-5-[(pyridin-3-yl)ethynyl]benzonitrile ([^18^F]-FPEB) to quantify mGluR5 binding in vivo in adults with autism vs. healthy controls using positron emission tomography (PET).

**Results:**

We identified significantly higher [^18^F]-FPEB binding potential in the postcentral gyrus and cerebellum of individuals with autism. There was a significant negative correlation between age and [^18^F]-FPEB binding potential in the cerebellum but not in the postcentral gyrus. In the precuneus, [^18^F]-FPEB binding potential correlated positively with the lethargy subscale score for the Aberrant Behavioral Checklist (ABC). In cerebellum, there were significant negative correlations between [^18^F]-FPEB binding potential and ABC total score, ABC hyperactivity subscale score, and the ABC inappropriate speech subscale score.

**Conclusions:**

These novel findings demonstrate for the first time that mGluR5 binding is altered in critical brain areas of subjects with autism, suggesting abnormal glutamate signaling in these regions. Finally, the correlations between altered [^18^F]-FPEB binding potential in the cerebellum and precuneus suggest that some autistic symptoms may be influenced by abnormal glutamate signaling.

## Background

Autism is a severe neurodevelopmental disorder with a rising incidence of 14.7 per 1000 (1 in 68) in the United States [1]. Autism is characterized by impairments in social communicative behavior and patterns of repetitive behavior [2]. Due to the heterogeneous nature of autism, the identity of autism-specific biomarkers remains elusive. Nevertheless, much of the focus has been placed on investigating neurotransmitter systems in the brains of subjects with autism. One of them, glutamate is the primary excitatory neurotransmitter in brain and spinal cord and is important in brain development and neuroplasticity [3]. Multiple studies have demonstrated elevated levels of glutamate in plasma of both adults and children diagnosed with autism when compared with controls [4–6]. Furthermore, magnetic resonance spectroscopy (MRS) studies have identified higher levels of glutamate in multiple brain regions of children and adolescents with autism including the anterior cingulate cortex (ACC), left striatum, left cerebellar hemisphere, and left frontal lobe [6, 7]. In contrast, reduced levels of the main inhibitory brain neurotransmitter, gamma-aminobutyric acid (GABA), have been observed in multiple brain regions of subjects with autism including the temporal lobe (auditory cortex), primary motor cortex, and postcentral gyrus (somatosensory cortex) [8–10]. These findings suggest that there is an imbalance in excitatory/inhibitory neurotransmission in the brains of subjects with autism. This imbalance could potentially explain the impairments that define the core symptoms of autism [11].

Accumulating data has implicated metabotropic glutamate receptor 5 (mGluR5) in the pathology of autism spectrum disorders [12–14]. Postmortem experiments have demonstrated increased expression of dimerized and total mGluR5 in cerebellar vermis and superior frontal cortex [Brodmann Area 9 (BA9)] in children with autism [12, 13]. In vitro binding assays employing [^3^H]-labeled 3-methoxy-5-pyridin-2-ylethynylpyridine (MPEPy) in prefrontal cortex tissue homogenates from subjects with fragile X syndrome (FXS) vs. controls found a marginally significant increase in mGluR5 density (*p* < 0.058) in subjects with FXS [14]. These findings are important in light of proposed treatments for autism [15] as well as schizophrenia and affective disorders [16] via the modulation of mGluR5 activity. If successful, mGluR5 modulation may improve symptoms of psychiatric disorders including autism in patients who have not improved via currently available treatment modalities.

The current study represents the first time that positron emission tomography (PET) imaging has been used to determine mGluR5 binding in vivo in brains of adults with autism and controls, using [^18^F]-3-fluoro-5-[(pyridin-3-yl)ethynyl]benzonitrile ([^18^F]-FPEB), a potent, selective, and systemically active antagonist of mGluR5 [17]. We aimed to investigate if mGluR5 expression in vivo replicated the results obtained by postmortem experiments and whether these changes (if any) positively correlated with autism symptom severity.

## Methods

### Patient recruitment

All study procedures were approved by the Johns Hopkins University Institutional Review Board (IRB) and all enrolled study subjects signed and dated IRB-approved consent forms. Informed consent was obtained from all individual participants included in the study. People with autism (*n* = 6) were recruited from a sample of children who had previously completed separate investigations at the Kennedy Krieger Institute (Baltimore, MD). Healthy control volunteers (*n* = 3) were recruited from the surrounding community via IRB-approved advertisements. Two historic controls, who had previously participated in a PET study conducted at Johns Hopkins University (JHU) were included. One of the historic controls could not be reached to complete new bloodwork and the Structured Clinical Interview for DSM-IV Axis I disorders – Clinician Version.

### Patient screening

All subjects were first screened using an IRB-approved telephone script to determine if they met the inclusion and exclusion criteria for the study. Additionally, all potential study subjects provided a complete medical and medication history, underwent a physical exam, vital signs, laboratory tests, and a 12-lead electrocardiogram (ECG). All study subjects (except for one historical control) underwent the Structured Clinical Interview for DSM-IV Axis I disorders – Clinician Version (SCID-CV) [18] in order to determine if they presented with psychiatric disorders. Autistic symptoms were determined by scores on the Autism Diagnostic Observation Schedule (ADOS) [19], the Autism Diagnostic Interview-Revised (ADI-R) [20], Autism Spectrum Screening Questionnaire (ASSQ) [21], the Clinical Global Impression (CGI) [22], the Aberrant Behavior Checklist (ABC) [23], the Lifetime Social Communication Questionnaire (SCQ) [24], and the Global Assessment of Functioning (GAF) [25, 26]. ADI-R and ADOS scores were obtained when the subjects with autism were children and were provided by the Kennedy Krieger Institute. SCID-5-CV, ASSQ, GCI, ABC, and GAF scores were obtained during patient screening.

Inclusion criteria for people with autism included: 1) male or female subjects, 18-35 years old; 2) previous diagnosis of autism spectrum disorder based on ADI-R and ADOS criteria; 3) weight of at least 100 lbs. (45.4 kg); 4) absence of other major serious, current medical, psychiatric, or neurologic issues other than autism and its comorbid deficits (i.e., seizure disorder, intellectual impairment). Subjects with comorbid diagnoses of attention deficit hyperactivity disorder (ADHD), obsessive compulsive disorder (OCD), or anxiety disorder were not excluded as there are high rates of comorbidity of these conditions in individuals diagnosed with autism [27, 28]; 5) provision of informed consent for testing from subject or an authorized decision maker. Inclusion criteria for healthy controls included the following: 1) male and female subjects, aged 18-35 years old, in good physical health; 2) have clinical laboratory test results within the reference ranges for the population or results within acceptable deviations that are not considered by the investigators to be clinically significant; 3) absence of other serious, current comorbid psychiatric disorders as determined by the SCID-CV; and 4) provision of written informed consent and ability to comply with the study restrictions. Exclusion criteria for people with autism and healthy controls were as follows: 1) change in behavioral treatments or life change such as change in residence, work site, or community service provider (within the past month); 2) pregnant or lactating women; 3) diagnosis of Tourette syndrome, FXS, or Rett syndrome; 4) patients with significant self-injury or with severity requiring inpatient treatment; 5) concurrent other psychiatric illness including substance abuse, or severe systemic disease based on history and physical exam; 6) laboratory tests with clinically significant abnormalities; 7) prior participation in other research protocols or clinical care in the last year such as radiation exposure that would exceed the annual limits; 8) suffer from claustrophobia and would be unable to undergo magnetic resonance imaging (MRI) or PET scanning; 9) implanted or embedded metal objects, prostheses, or fragments in the head or body that would present a risk during the MRI scanning procedure; 10) clinically significant abnormal MRI; 11) positive human immunodeficiency virus (HIV) test; 12) alcohol consumption within 48 h before the PET scan; 13) currently a user of any illicit drugs or alcohol abuse, or has a positive drug screen; 14) BMI of > 40 kg/m^2^ 15) use of prescription stimulants in the two days before the PET scan; 16) currently on medications that affect the glutamate system.

### MRI

Subjects, who met the enrollment criteria following the initial screening assessments, underwent an MRI scan with a set format of structural sequences including a spoiled gradient recalled (SPGR) acquisition sequence (124 slices with image matrix 256 × 256, pixel size 0.93 × 0.93 mm, slice thickness 1.5 mm) imaging for a three-dimensional anatomical data set of the brain [29] using a 3-T Magnetom Trio scanner (Siemens Medical Solutions). The MRI scans were obtained on each subject in order to co-register PET and MRI images for analysis of PET data.

### ^18^F-FPEB preparation

^18^F-FPEB was prepared at high specific activity as previously described [17].

### PET scan

All PET scans were performed at the JHU PET Center using a second-generation, High-Resolution Research Tomograph (HRRT; CPS Innovations, Inc.) (2 mm axial resolution). A venous catheter was placed prior to the scan for radiotracer injection. Approximately 10 min before ^18^F-FPEB injection, a transmission scan was acquired for attenuation correction. Subjects were administered intravenously approximately 185 megabecquerel (MBq) [5 mCi (mCi)] [^18^F]-FPEB dose in saline. Dynamic PET scans of the brain began immediately after [^18^F]-FPEB administration and images were acquired for 90 min after tracer administration.

### Statistical analysis

Quantitative analysis of the non-displaceable binding potential (BP_ND_) of [^18^F]-FPEB was obtained to measure the density of the mGluR5 availability (B_max_) in selected brain regions. Cerebellar white matter was defined as the reference tissue for [^18^F]-FPEB. We determined volumes of interest (VOIs) based on co-registered MRI and PET scans for analysis with a multilinear reference tissue, two parameter model. Brain regional BP_ND_s were obtained. Descriptive and analytical methods for small samples were employed. To determine differences in binding potential of [^18^F]-FPEB between individuals with autism vs. controls, two-tailed student’s t-tests were run for each of the brain regions with significance set at *p* < 0.05. Two-tailed Pearson correlations were calculated to determine potential relationships between [^18^F]-FPEB binding potential and measures of autistic symptoms and scores on psychometric tests in subjects with autism.

## Results

A total of six individuals with autism were used for data analysis. Three control individuals were included, two of whom had previously been scanned with [^18^F]-FPEB as part of an earlier study. The mean age of controls was 27 ± 3.61 and the mean age of subjects with autism was 20 ± 2.10 (*p* < 0.0067). All subjects with autism and controls were male. With regard to race, for the controls one was white, one was African American, and one was Asian American. For the subjects with autism five were white and one was Asian American. Table 1 summarizes demographic data and Table 2 summarizes mean scores for the study subjects on measures of autistic pathology.

**Table 1:** Demographics of study subjects

	Control	Autism	*P*
Age	27 ± 3.61	20 ± 2.10	*0.0067*
Sex	3 M	6 M	–
Race	1 W, 1 A, 1AA,	5 W, 1A	–

*A* Asian, *AA* African American, *M* male, *W* white

Italicized value represents a statistically significant finding

**Table 2:** Measures of autism symptoms of study subjects

Measure	Range of scores	Autism cutoff score	Score
Autism Diagnostic Observation Schedule (ADOS)			
Language and communication total	(0-12)	> 3	4.50 ± 1.87
Reciprocal social interaction total	(0-18)	> 6	8.00 ± 3.54
Language and communication and reciprocal social interaction total	(0-30)	> 10	12.50 ± 3.90
Autism Diagnostic Interview-Revised (ADI-R)			
Reciprocal social interaction total	(0-32)	> 10	15.83 ± 5.12
Communication total	(0-26)	> 8	12.67 ± 4.08
Restricted, Repetitive and stereotyped behavior total	(0-16)	> 3	5.33 ± 1.86
Total	(0-70)	NA	34.00 ± 7.96
Autism Spectrum Screening Questionnaire (ASSQ)			
Total	(0-54)	> 17	17.17 ± 7.88
Aberrant Behavior Checklist (ABC)			
Irritability total	(0-45)	NA	3.50 ± 5.68
Lethargy total	(0-48)	NA	10.50 ± 8.36
Stereotypy total	(0-21)	NA	1.50 ± 1.22
Hyperactivity total	(0-48)	NA	7.83 ± 6.01
Inappropriate speech total	(0-12)	NA	1.83 ± 1.72
Total	(0-174)	NA	25.17 ± 14.27
Clinical Global Impression			
Severity of illness	(0-7)	NA	3.50 ± 1.05
Global Improvement	(0-7)	NA	3.17 ± 1.33
Efficacy index	(0-16)	NA	9.33 ± 4.03
Lifetime Social Communication Questionnaire (SCQ)			
Total for verbal children	(0-39)	> 15	16.17 ± 3.92
Global Assessment of Functioning (GAF)			
Total	(0-90)^a^	NA	69.83 ± 9.15

*NA* not applicable; ^a^For this scale, a higher score indicates better functioning

**Fig. 1:**
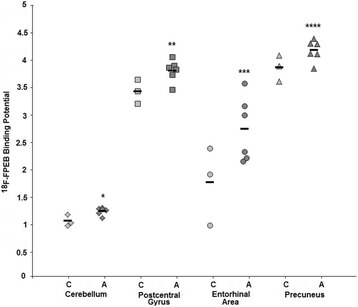
[^18^F]-FPEB binding potential in selected brain regions. Scatter plot showing [^18^F]-FPEB binding potential in cerebellum, postcentral gyrus, entorhinal area, and precuneus of subjects with autism vs. controls. A, autism; C, control; *, *p* < 0.016; **, *p* < 0.036; ***, *p* < 0.065; ****, *p* < 0.071

[^18^F]-FPEB binding potential was measured in 21 brain regions, all of which are known to express mGluR5 [17, 30–37] and have previously been implicated in the pathology of autism [38–46]. We identified significantly elevated [^18^F]-FPEB binding potential in cerebellum (*p* < 0.016) and postcentral gyrus (*p* < 0.036), indicating increased mGluR5 binding in these brain regions (Table 3, Fig. 1). Moreover, we identified trends towards significant elevation of [^18^F]-FPEB binding potential in the entorhinal area (*p* < 0.065) and the precuneus (*p* < 0.071) (Table 3, Fig. 1). Because age was significantly different between controls and subjects with autism, Pearson correlations were run to analyze this demographic measure for an effect on [^18^F]-FPEB binding potential. For cerebellum, there was a significant negative correlation between age and [^18^F]-FPEB binding potential (*r* = − 0.68, *p* < 0.042). However, correlations were not significant for postcentral gyrus (*r* = − 0.43, *p* < 0.25), precuneus (*r* = − 0.36, *p* < 0.34) or entorhinal area (*r* = − 0.38, *p* < 0.32).

**Table 3:** ^18^F-FPEB binding potential throughout the brain as revealed by positron emission tomography (PET)

Region	Control	Autism	*t*-value	*P*
Amygdala	3.93 ± 0.64	3.69 ± 0.57	0.58	0.58
Caudate nucleus	4.28 ± 0.47	4.56 ± 0.62	0.67	0.52
Cerebellum	1.07 ± 0.10	1.25 ± 0.068	3.17	*0.016*
Cingulate	4.33 ± 0.43	4.42 ± 0.23	0.42	0.68
Entorhinal area	1.78 ± 0.71	2.75 ± 0.59	2.19	*0.065*
Frontal lobe	4.11 ± 0.39	4.28 ± 0.22	0.87	0.41
Fusiform gyrus	3.86 ± 0.49	3.69 ± 0.52	0.47	0.65
Globus pallidus	1.12 ± 0.14	1.28 ± 0.15	1.58	0.16
Hippocampus	3.74 ± 0.67	3.66 ± 0.52	0.21	0.84
Insula	4.58 ± 0.62	4.49 ± 0.51	0.22	0.83
Occipital lobe	3.22 ± 0.39	3.35 ± 0.15	0.79	0.46
Paracentral gyrus	2.89 ± 0.44	3.36 ± 0.32	1.83	0.11
Parahippocampus	3.25 ± 0.09	3.55 ± 0.40	1.22	0.26
Parietal lobe	3.93 ± 0.36	4.10 ± 0.14	1.08	0.32
Precentral gyrus	3.55 ± 0.38	3.86 ± 0.12	1.89	0.10
Precuneus	3.87 ± 0.24	4.19 ± 0.20	2.13	*0.071*
Postcentral gyrus	3.44 ± 0.22	3.82 ± 0.20	2.60	*0.036*
Putamen	3.90 ± 0.54	4.18 ± 0.33	0.91	0.39
Temporal lobe	4.23 ± 0.57	4.23 ± 0.30	0.02	1.00
Thalamus	2.43 ± 0.28	2.50 ± 0.29	0.37	0.72
Ventral striatum	4.75 ± 0.66	5.10 ± 0.50	0.89	0.40

Italicized values represent significant or near-significant findings

**Fig. 2:**
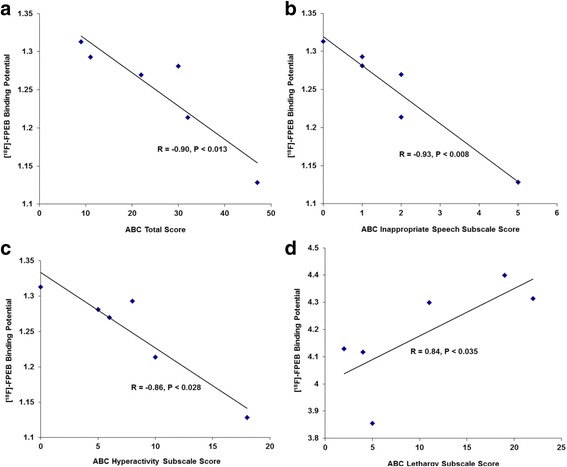
Correlations between [^18^F]-FPEB binding potential and scores on psychometric tests. Scatter plots showing correlations between [^18^F]-FPEB binding potential and ABC total score (**a**), ABC inappropriate speech subscale score (**b**), ABC hyperactivity subscale score (**c**), and ABC lethargy subscale score (**d**) in subjects with autism. **a**, **b**, and **c** [^18^F]-FPEB binding potentials are from cerebellum; **d** [^18^F]-FPEB binding potential is from precuneus

In cerebellum, there were significant negative correlations between [^18^F]-FPEB binding potential and ABC total score (*r* = − 0.904, *p* < 0.013), ABC hyperactivity subscale score (*r* = − 0.861, *p* < 0.028), and the ABC inappropriate speech subscale score (*r* = − 0.928, *p* < 0.008) (Fig. 2). In precuneus, there was a significant positive correlation between [^18^F]-FPEB binding potential and ABC lethargy subscale score (*r* = 0.843, *p* < 0.035) (Fig. 2).

## Discussion

We observed significantly elevated [^18^F]-FPEB binding potential in two brain regions of males with autism: the cerebellum (in its entirety) and the postcentral gyrus. We also observed trends for significantly elevated [^18^F]-FPEB binding potential in the precuneus and the entorhinal area. In subjects with autism, we identified significant, negative correlations between [^18^F]-FPEB binding and ABC total score, ABC hyperactivity subscale score, and ABC inappropriate speech subscale score in cerebellum. We also identified one significant positive correlation between [^18^F]-FPEB binding and ABC lethargy subscale score in precuneus.

Increased mGluR5 expression has previously been observed in cerebellar vermis and BA9 of children with autism [12, 13]. These findings suggest pathologic activation of mGluR5 which may be prenatal or early postnatal. The observed PET data of elevated [^18^F]-FPEB binding potential in young adults with autism supports this hypothesis. In contrast, a recent study found reduced mGluR5 immunoreactivity in the dorsolateral prefrontal cortex (DLPFC) of subjects with autism [47]. However, this finding may be due to the mixing of adult and child subjects. Additionally, the authors used immunohistochemistry and not western blotting or imaging, which may account for possible differences between their results and ours. As shown in the comparison between age of study subjects and [^18^F]-FPEB binding potential, there was a significant negative correlation between age and [^18^F]-FPEB binding potential in the cerebellum, while there was no effects in the postcentral gyrus, precuneus, or the entorhinal area. These results are likely impacted by low sample size. Thus, repetition of these experiments involving a larger sample is needed to determine the effect of age on [^18^F]-FPEB binding potential. The negative correlations between [^18^F]-FPEB binding potential and ABC total score as well as hyperactivity and inappropriate speech subscale scores in the cerebellum provide some evidence that increased mGluR5 binding in autism is not associated with greater symptom severity. The one positive correlation between [^18^F]-FPEB binding and the ABC-lethargy subscore in precuneus is intriguing given that the precuneus is a principal component of the default mode network (DMN). The DMN is active when the individual is engaged in internally focused tasks including social cognition, a major impairment in individuals with autism [48]. As reviewed by Padmanabhan et al. [48], there are structural and functional abnormalities in the DMN of individuals with autism. Excitatory/inhibitory imbalance as indicated by changes in levels of glutamate and/or GABA could potentially impact functioning of the DMN and autistic symptomatology including lethargy as measured by the ABC. Further studies are needed however, to confirm these findings.

The four brain regions that showed significant increased or trends for increased [^18^F]-FPEB binding potential are involved in important cognitive domains that are impaired in autism including motor control, facial recognition, and memory [49–55]. The cerebellum plays a crucial role in learning and control of action through sensorimotor adaptation of signals in motor, premotor, and prefrontal cortex [51, 54]. The postcentral gyrus contains the primary somatosonsory cortex and is thereby crucial to motor control and learning and facial recognition [50]. The precuneus is located in the medial parietal cortex and maintains functional connection with the prefrontal cortex [56]. PET studies have shown precuneus activation during episodic memory tasks [52, 53]. The entorhinal cortex is the main region of interaction between the hippocampus and neocortical regions due to its reciprocal connections between these two areas and is involved with memory [55].

Studies have previously demonstrated functional and morphological changes in all four of these brain regions in people with autism [38–40, 42]. A number of motor learning studies have suggested that people with autism show a bias towards reliance on proprioceptive (as opposed to visual) feedback [57–59], with evidence that abnormalities in the sensorimotor regions of the cerebellum that may contribute to this bias. Our findings provide additional support for abnormalities in these somatosensorimotor-cerebellar circuits, crucial to development of skilled actions necessary in social and communicative behavior. The postcentral gyrus of subjects with autism has been shown to display reduced cortical thickness and reduced gray matter concentration when compared with controls [42]. Cheng et al. [40], found that gray matter volume was reduced in postcentral gyrus while gray matter volume is increased in cerebellum in subjects with autism vs. controls using voxel-based morphometric analysis. An analysis of whole-brain voxel-based unbiased resting state functional connectivity found reduced connectivity in multiple brain regions including left and right precuneus and left and right postcentral gyrus of subjects with autism [39]. Moreover, functional connectivity changes in these two regions were significantly associated with ADOS severity scores. Morphological changes in the cerebellum of subjects with autism have been identified including: 1) altered Purkinje cell density; 2) abnormalities in deep cerebellar nuclei; and 3) changes in total cerebellar volume [51]. These changes may contribute to motor and cognitive deficits associated with autism. Small tightly packed neurons have been consistently found in the entorhinal cortex of subjects with autism [38]. These anatomical changes, coupled with potential changes in glutamate signaling, likely contribute to impaired cognitive domains in autism.

Strengths of the current study include: 1) high resolution tomograph (the HRRT PET scanner is the highest resolution dedicated brain PET scanner available) with kinetic modeling; 2) the use of state of the art technology and one of the most optimal PET radiotracers for mGluR5 human brain imaging; and 3) carefully screened and characterized subjects. Limitations of the current study include: 1) small sample size; 2) differences in racial/ethnic diversity; 3) the age differences between subjects with autism vs. controls; and 4) the lack of inclusion of both sexes. Of the limitations, small sample size is important and as we have mentioned previously, further studies using a larger sample size are needed to confirm our results. The significant difference in age between subjects with autism vs. controls was an important limitation, which moreover had an effect on [^18^F]-FPEB binding potential in the cerebellum, but not the postcentral gyrus, precuneus, or entorhinal area. This may be an artifact that is tied to small sample size. In our previous studies of mGluR5 expression in children and adults with autism vs. controls, analysis of confounds did not find an effect of age on our results for cerebellar vermis [13]. The differences in racial/ethnic diversity between groups are a limitation that is also partially tied to small sample sizes. It is thus, difficult to determine whether these differences have an impact on [^18^F]-FPEB binding potential. Finally, while autism is more prevalent in males than in females [60], further studies should include females as well to ensure that the observed differences are not sex-specific.

## Conclusions

The current study represents the first measurement of mGluR5 concentration in vivo in young adults with autism. Our findings partially validated our hypotheses in that some brain regions displayed significant elevations in elevated [^18^F]-FPEB binding potential and there was one significant positive correlation between [^18^F]-FPEB binding potential and a symptom of autism. Based on this pilot study, further experiments measuring mGluR5 binding in individuals with autism are warranted. mGluR5 may prove an important target of therapeutic intervention in autism spectrum disorders.

## Abbreviations

[^18^F]-FPEB: [^18^F]-3-fluoro-5-[(pyridin-3-yl)ethynyl]benzonitrile
ABC: Aberrant Behavior Checklist
ACC: Anterior cingulate cortex
ADHD: Attention deficit hyperactivity disorder
ADI-R: Autism Diagnostic Interview-Revised
ADOS: Autism Diagnostic Observation Schedule
ASSQ: Autism Spectrum Screening Questionnaire
BA9: Brodmann area 9
B_max_: The ratio of the available receptor density
BMI: Body mass index
BP_ND_: Non-displaceable binding potential
CGI: Clinical Global Impression
DLPFC: Dorsolateral prefrontal cortex
DSM: Diagnostic and Statistical Manual
ECG: Electrocardiogram
FXS: Fragile X syndrome
GABA: Gamma-aminobutyric acid
GAF: Global Assessment of Functioning
HIV: Human immunodeficiency virus
HRRT: High-Resolution Research Tomograph
IRB: Institutional review board
JHU: Johns Hopkins University
K_D_: Radioligand equilibrium dissociation constant
MBq: Megabecquerel
mCi: Millicurie
mGluR5: Metabotropic glutamate receptor 5
MRI: Magnetic resonance imaging
MRS: Magnetic resonance spectroscopy
OCD: Obsessive compulsive disorder
PET: Positron Emission Tomography
SCID-CV: Structured Clinical Interview for DSM-IV Axis I disorders – Clinician Version
SCQ: Lifetime Social Communication Questionnaire
SPGR: Spoiled gradient recalled
